# Comparison of In Vitro and In Vivo Results Using the GastroDuo and the Salivary Tracer Technique: Immediate Release Dosage Forms under Fasting Conditions

**DOI:** 10.3390/pharmaceutics11120659

**Published:** 2019-12-07

**Authors:** Maximilian Sager, Philipp Schick, Magdalena Mischek, Christian Schulze, Mahmoud Hasan, Marie-Luise Kromrey, Hassan Benameur, Martin Wendler, Mladen Vassilev Tzvetkov, Werner Weitschies, Mirko Koziolek

**Affiliations:** 1 Department of Biopharmaceutics and Pharmaceutical Technology, Institute of Pharmacy, University of Greifswald, 17489 Greifswald, Germany; sager.max@gmail.com (M.S.); mirko.koziolek@uni-greifswald.de (M.K.); 2 Department of Pharmaceutical Biology, Institute of Pharmacy, University of Greifswald, 17489 Greifswald, Germany; 3 Department of Pharmaceutical Chemistry, Future University in Egypt, Cairo 11835, Egypt; 4 Department of Clinical Pharmacology, University Medicine Greifswald, 17489 Greifswald, Germany; 5 Department of Diagnostic Radiology and Neuroradiology, University Medicine Greifswald, 17489 Greifswald, Germany; 6 Lonza R&D Center of Excellence, 67400 Illkirch-Graffenstaden, France; 7 Institute of Mathematical Stochastics, Faculty of Mathematics, Otto-von-Guericke University, 39106 Magdeburg, Germany

**Keywords:** dissolution methods, biorelevant in vitro model, clinical study, salivary tracer technique, GastroDuo

## Abstract

The fasted state administration of immediate release (IR) dosage forms is often regarded as uncritical since physiological aspects seem to play a minor role for disintegration and drug release. However, recent in vivo studies in humans have highlighted that fasted state conditions are in fact highly dynamic. It was therefore the aim of this study to investigate the disintegration and drug release behavior of four different IR formulations of the probe drug caffeine under physiologically relevant conditions with the aid of the GastroDuo. One film-coated tablet and three different capsule formulations based on capsule shells either made from hard gelatin or hydroxypropylmethyl cellulose (HPMC) were tested in six different test programs. To evaluate the relevance of the data generated, the four IR formulations were also studied in a four-way cross-over study in 14 healthy volunteers by using the salivary tracer technique (STT). It could be shown that the IR formulations behaved differently in the in vitro test programs. Thereby, the simulated parameters affected the disintegration and dissolution behavior of the four IR formulations in different ways. Whereas drug release from the tablet started early and was barely affected by temperature, pH or motility, the different capsule formulations showed a longer lag time and were sensitive to specific parameters. However, once drug release was initiated, it typically progressed with a higher rate for the capsules compared to the tablet. Interestingly, the results obtained with the STT were not always in line with the in vitro data. This observation was due to the fact that the probability of the different test programs was not equal and that certain scenarios were rather unlikely to occur under the controlled and standardized conditions of clinical studies. Nonetheless, the in vitro data are still valuable as they allowed to discriminate between different formulations.

## 1. Introduction

It is generally assumed that the fasted state administration of immediate release (IR) drug products results in less variable drug plasma concentrations as compared to fed state administration. This conception is based on two main assumptions: (1) drug release from IR formulations is fast and, (2) the physiological parameters (e.g., gastric emptying, gastric pH) of the human gastrointestinal (GI) tract which are important for oral drug delivery are less variable. For this reason, drug release from IR formulations under fasted state conditions is often not assessed by use of biorelevant dissolution test methods.

After oral administration, several physiological factors such as luminal fluid volumes present in stomach and small intestine, mechanical stresses resulting from peristalsis, luminal pH values, and temperature can be relevant for drug release from IR formulations. In particular, the gastric emptying kinetics of the coadministered fluid is crucial since it controls the delivery of dissolved and undissolved drug to the small intestine—the main site of absorption. A deeper understanding of the physiological variability of these parameters under the controlled conditions of bioequivalence and bioavailability studies (BE/BA) conducted in accordance with Food and Drug Administration( FDA) and European Medicines Agency (EMA) guidelines [1], Ref. [2] was gained in recent years through different in vivo investigations that aimed at characterizing the physiological conditions in the stomach and the small intestine [3,4,5,6]. These studies revealed that the conditions in the human GI tract can be highly variable and in particular, gastric emptying was shown to be largely dynamic [5]. Recently, Grimm et al. investigated the inter and intrasubject variability in gastric emptying [6].

Gastric emptying causes dynamic changes of the fluid volumes present in the fasted stomach. Since this medium represents the medium available for drug dissolution, the kinetics of disintegration and dissolution have a direct impact on intestinal drug concentrations which in turn are relevant for drug absorption (e.g., passive diffusion, capacity of uptake transporters) as well as for intestinal metabolism and/or elimination (e.g., efflux transporters). For instance, a delayed disintegration of a formulation may lead to higher drug concentrations in the stomach because of reduced gastric fluid volumes present at later time points. As a consequence, drug concentrations in the small intestine will be higher. Thus, variable drug release in the stomach can lead to variable drug concentrations in the small intestine, which potentially affect drug absorption.

In order to reduce the variability in drug release and to develop robust and reliable IR formulations, biorelevant in vitro test methods can be applied to simulate the dynamic situation in the human stomach. The application of compendial dissolution tests does typically not allow mimicking the complex luminal environment to which oral drug products are exposed in reality. In the last years, several groups have made great efforts to overcome this issue by developing biorelevant dissolution media as well as in vitro models of higher physiological relevance [7]. These range from rather simple (e.g., transfer model, BioGIT) to more complex models (e.g., Dynamic Gastric Model, TNO TIM-1) [8,9,10]. Depending on the focus of the dissolution experiment, each model has specific advantages and disadvantages [7,11].

With respect to gastric emptying and motility, there is a clear need for improved in vitro test systems that enable the consideration of the variability of the physiological conditions in the stomach. In the past, we have already demonstrated the importance of gastric emptying for the drug plasma concentrations of two different IR formulations containing N-acetylcysteine by use of the Dynamic Open Flow-Through Test Apparatus [12]. In this work, we describe the design and application of the GastroDuo as an optimized biorelevant in vitro tool for the investigation of drug release in the stomach. The GastroDuo was initially introduced by Schick et al. as a valuable tool to in vitro simulate crucial parameters of the human stomach [13]. The primary aim of this work was to assess the biorelevance of the results obtained with the GastroDuo by comparing in vitro and in vivo data on drug release from four different IR formulations containing caffeine as a probe drug. For this purpose, drug release from the four IR formulations was first investigated in vitro by using compendial dissolution methods and the GastroDuo. Subsequently, we performed an in vivo study with 14 healthy volunteers, in which the salivary tracer method was used to determine the in vivo disintegration behavior of the four formulations [14]. The secondary aim of this study was to elaborate on the effect of different physiological factors on the drug release behavior of three different capsule shells and a film-coated tablet.

## 2. Materials and Methods

### 2.1. Materials

Caffeine, croscarmellose, and lactose monohydrate were supplied by Caelo (Hilden, Germany). Polyvinylpyrrolidone (PVP) 90, magnesium stearate, silica dioxide, and hydroxypropyl methylcelluose K4M (HPMC) were purchased from Fagron (Barsbüttel, Germany).

Water HiPerSolv CHROMANORM LC-MS grade (VWR international, Fontenay-sous-Bois, France), Methanol HiPerSolv CHROMANORM LC-MS grade (VWR international, Fontenay-sous-Bois, France), Formic acid (Merck KaA Gmbh, Darmstadt, Germany), Acetonitrile HiPerSolv CHROMANORM LC-MS grade (VWR international, Fontenay-sous-Bois, France), ammonium acetate (Merck KaA Gmbh, Darmstadt, Germany), completely desalinated water (based on tap water, Stadtwerke Greifswald, Germany, prepared by double reverse osmosis).

### 2.2. Dosage Forms Investigated

#### 2.2.1. Immediate Release Tablets

Immediate release tablets were compressed from a powder mixture composed of caffeine (7% *w/w*), croscarmellose (12% *w/w*), and lactose (81% *w/w)*, which was granulated by using a solution (4.5% *v/v*) of PVP-90. Magnesium stearate and silica dioxide were added directly before tableting. In the end, biconvex tablets (14 × 6 mm) with a total weight of 350 mg were prepared on a rotary tablet press (Riva Piccola, Hampshire, UK) at a compression force of approximately 6.7 kN. The tablets showed a mean crushing strength of 87 N (*n* = 10) in a range from 76 to 100 N. Subsequently, an HPMC coating of 5% (weight gain) was applied onto the tablets with the aid of a drum coater (Glatt, Germany). 

#### 2.2.2. Capsules

Three different capsule shells (size 0) were used in this study (Table 1). All capsules were directly filled with 350 mg of the nongranulated powder mixture that was also used for tableting. Hard gelatin and Vcaps^®^ plus capsules were purchased from Capsugel (Morristown, NJ, USA). Quali-V^®^ capsules were supplied by Qualicaps (Yamatokoriyama Nara, Japan).

### 2.3. In Vitro Investigations

The in vitro investigations were performed by using the compendial USP II paddle apparatus and a novel biorelevant dissolution test device, the GastroDuo.

#### 2.3.1. Compendial Dissolution

The USP II paddle apparatus (PharmaTest DT70, Erweka, Heusenstamm, Germany) mainly served as a reference in this work. All dosage forms were tested in 900 mL of simulated gastric fluid (SGF) pH 1.2 at a temperature of 37 °C and a stirring speed of 75 rpm. Drug concentrations inside the vessels were determined with a spectrophotometer (Cary 60, Agilent, Santa Clara, CA, USA) that was equipped with a 16-channel fiber optic system. The detection wavelength was 272 nm.

#### 2.3.2. GastroDuo 

*Construction and Setting*

The GastroDuo is a biorelevant dissolution test device that combines the different advantages of the different in vitro models (i.e., Dissolution StressTest device, Dynamic Open Flow-Through Test Apparatus, and Fed Stomach Model) that were previously developed in our group [12,15,16,17]. The GastroDuo is able to simulate certain physiological aspects of the stomach and the small intestine. This includes gastric emptying kinetics, luminal pH, and temperature profiles as well as a realistic simulation of pressures arising during GI transit due to motility. In order to enable the in vitro simulation of these factors, several modifications were made to the construction of the Fed Stomach Model [16]. 

**Figure 1:**
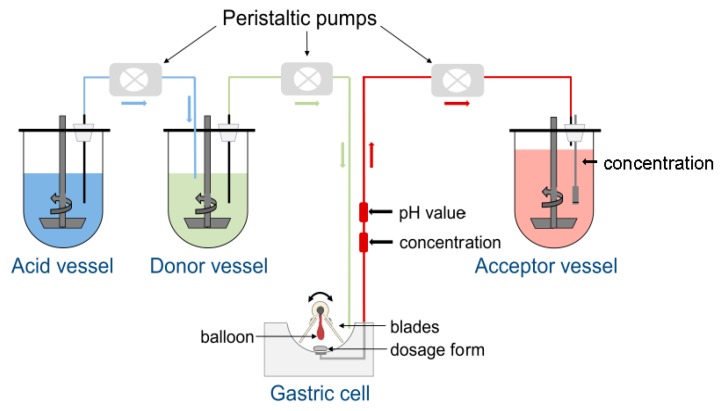
Schematic presentation of the experimental setup for the GastroDuo.

As can be seen from Figure 1, the central element of the GastroDuo is the gastric cell, which represents a flow-through cell with a maximum volume of 50 mL. The gastric cell is perfused with medium from the donor vessel. The pH value of this donor medium can be further adjusted by the addition of acidic media from the acid vessel to allow the simulation of different pH profiles in the gastric cell. To simulate realistic gastric emptying kinetics, the medium from the gastric cell is pumped with defined rates into the acceptor vessel, which contains a medium that allows complete drug dissolution. During the experiment, the volume inside the gastric cell is kept constant. A proper temperature control is ensured by the surrounding water bath and the low wall thickness (approximately 0.75 mm) of the gastric cells that are made from Vivak^®^ (PET-G, Bayer, Kaiser-Wilhelm-Allee 1, 51373 Leverkusen, Germany). 

For the investigation of drug release, a formulation is placed in the center of the gastric cell. Two comb-like blades that are connected to a central axis enable the simulation of formulation movement in the stomach as well as mixing. A balloon located between the blades is directly connected to the central axis and can be inflated with different pressures to simulate mechanical stresses acting on the formulation. The magnitude of these stresses is based on recent in vivo investigations with the SmartPill^®^ [3]. The drug concentrations are constantly measured at two positions: (1) at the outlet of the gastric cell (in-line) to assess changes of drug concentration in the fluid emptied from the simulated gastric compartment and, (2) in the acceptor vessel to determine the sum of dissolved and undissolved drug substance emptied from the gastric cell. In addition, pH and temperature are constantly monitored in the outflow of the gastric cell with the aid of a pH electrode (InLab^®^ Expert Pro, Mettler Toledo, Switzerland) and a temperature sensor (Board: USB-µPIO TEMP12, Abacom; Software: RealView, Electronics Software; Sensor: DS18B20). In total, the GastroDuo consists of three gastric cells so that three experiments can be conducted at the same time. In the Supplementary Material, a video can be found that shows the functional principle of the GastroDuo.

*Test Programs*

The GastroDuo allows the simulation of different conditions and events. In this study, the effect of different rates of gastric emptying, different pH and temperature profiles, and pressure events of physiological magnitude were studied. In order to reflect the physiological variability of these factors in the fasted stomach, six test programs (A–F) were defined. The ranges of the physiological parameters that are covered by these test programs were based on recent in vivo studies performed with SmartPill and MRI [3,5,6].

All test programs consisted of a gastric transit part with a defined duration and a subsequent artificial part that was used to flush out all residues of the drug from the gastric cell. The gastric transit part always ended with a simulated gastric emptying pattern that included an increased flow rate of 20 mL/min, three pressure events of 300 mbar and three fast movements of the blades. This gastric emptying event simulated the complete emptying of the stomach by a migrating motor cycle (MMC) phase III contraction wave (“housekeeping wave”). At the beginning of the experiments, the gastric cells were filled with 25 mL of pure deionized water. The first order like gastric emptying of the coadministered water was simulated by six decreasing flow rate “steps”. In each experiment, a cumulative volume of 240 mL was perfused through the gastric cell, which was equal to the volume of water given in the in vivo study. The poor mixing of the stomach was simulated by a slow movement of the blades which was performed every 3 min.

**Figure 2:**
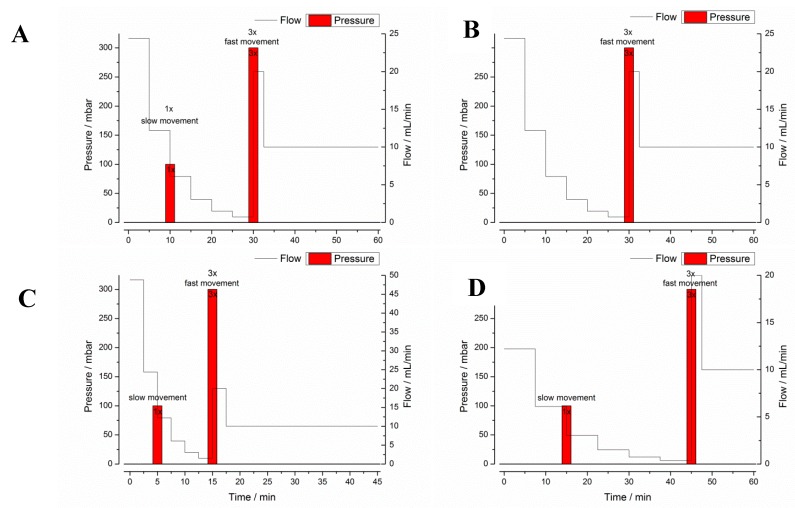
Graphical illustration of the GastroDuo test programs (**A**–**D**) with pressure and flow rates over time. Text boxes additionally indicate slow or fast movements.

**Figure 3:**
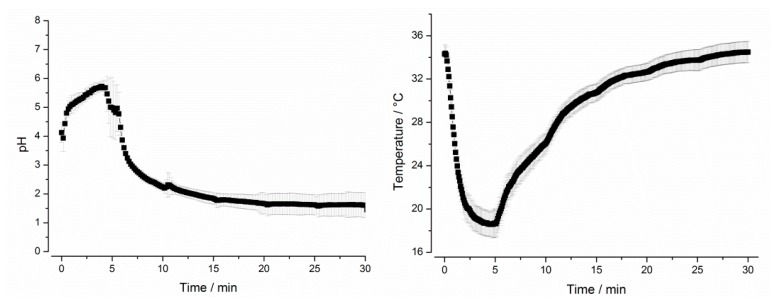
*Left*: pH profile simulated in test program A. *Right*: Temperature profile simulated in test program A (mean ± SD, *n* = 6).

Program A was designed to represent the average conditions in the stomach after administration of 240 mL of tap water and was based on mean values in terms of gastric emptying, motility, pH, and temperature. All other programs (B–F) simulated the extremes of these particular parameters. Test program A simulated a gastric transit time (GTT) of 30 min and a small stress event after 10 min, whereas test program B had no such stress event after 10 min. Test programs C and D were designed to simulate short (15 min, C) and long (45 min, D) GTT and, therefore, different hydrodynamic stresses. Thus, the test programs A–D simulated the variability of gastric emptying and gastric peristalsis (Figure 2). In all these programs, dynamic temperature and pH profiles were simulated. The simulated profiles were based on recent in vivo data, which were obtained by administering telemetric capsules in the fasted state together with 240 mL of tap water [3]. These measurements clearly showed that both parameters, pH and temperature, follow a distinct profile in the fasted stomach. As can be seen from Figure 3, the GastroDuo enabled the simulation of specific pH and temperature profiles. Whereas the pH value of the simulated gastric medium was increased for a short time, the temperature fell to values below 20 °C.

The test programs E and F served as reference programs to study the effect of temperature and pH changes on the drug release behavior of the four IR formulations. These programs were identical to test program A in terms of duration, emptying kinetics, and motility events (Figure 2) and differed only in terms of temperature (E) or pH (F). In program E, the temperature was kept constant at 37 °C (program E) and in program F, the pH was constantly at pH 1.2.

### 2.4. Determination of In Vitro Disintegration Parameters

Drug concentrations in the outflow of the gastric cells and the acceptor vessels were determined with a spectrophotometer (Cary 60, Agilent) that was equipped with a 16-channel fiber optic system. The detection wavelength was 272 nm. 

On the basis of the drug concentrations measured in the outflow of the gastric cell, the initial capsule or tablet disintegration (iDT) was calculated to describe the in vitro disintegration behavior. The iDT was defined by the measurement of a caffeine concentration that exceeds the lower limit of quantification (LLOQ = 15 µg/mL).

### 2.5. In Vivo Study with the Salivary Tracer Method

An in vivo study based on the recently reported salivary tracer method was performed to investigate the disintegration behavior of the four IR formulations in the human GI tract. The procedure applied in this study followed the description of a recent publication “Low dose caffeine as a salivary tracer for the determination of gastric water emptying in fed and fasted state: A MRI validation study” [14].

#### 2.5.1. Study Design

In this study, 14 healthy volunteers of both sexes were included. The information about the characteristics of the study participants is provided in Table 2.

Written informed consent was obtained from every participant. The study was conducted in compliance with the “Declaration of Helsinki” (revised version from 2013, Fortaleza) and the “(Model) Professional Code for Physicians in Germany” (revised version from 2011, Kiel). A positive vote was given by the ethics committee of the University Medicine Greifswald for the study named “Nutzung von Speichelmarkern zur in vivo Charakterisierung von Arzneiformen” (registration date: 17 November 2017, registration number: BB 172/17). For every participant, insurance was concluded to all risks associated with participating. 

The study was conducted in a randomized four-way, cross-over design. Thus, all volunteers received the four formulations in a randomized manner on different days with a wash-out period of at least 48 h. After an overnight fast of at least 10 h, the volunteers had to take the dosage form in an upright position together with 240 mL of tap water (time point 0 min). Afterwards, the oral cavity was rinsed three times with around 100 mL of tap water without swallowing for cleaning purposes. Saliva samples were collected by drooling at the time points: −5, 3, 5, 7, 9, 11, 13, 15, 17, 19, 21, 26, 31, 36, 41, 51, 61 min plus every 15 min until 241 min.

#### 2.5.2. Preparation and Analysis of Saliva Samples

All saliva samples were stored at −80 °C immediately at the end of each study day until analysis. Saliva sampling and probe preparation was based mainly on the procedure described in the recent publication [14].

Determination of caffeine in the saliva samples was performed by use of an Agilent 1100 series HPLC system (Agilent Technologies, Waldbronn, Germany) coupled to a triple quadrupole mass spectrometer API4000 QTRAP (AB Sciex, Darmstadt, Germany) via the electrospray ionization source Turbo V™. The LC-MS/MS system was controlled by the validated Analyst 1.6 software (AB Sciex, Darmstadt, Germany).

Caffeine was separated from hydrophilic saliva components such as mucins and other glycoproteins by isocratic elution. The mobile phase consisted of a 50/50 (*v/v*)-mixture of ammonium acetate buffer (5 mM; pH 3.8) and methanol. The flow rate was set to 250 μL/min. The reversed-phase column XTerra^®^MS (C18, 3.5 µm, 2.1 × 100 mm; Waters, Dublin, Ireland) was tempered at 40 °C. The injection volume was 20 μL. 

The chromatographic flow was directed to a 0.5 µm filter device (PEEK, Supelco, Taufkirchen, Germany) to avoid particulate contamination. The HPLC was connected to the mass spectrometer interface (Turbo V™ ionization source) operated in the positive ion mode. The following gas parameters were used: temperature, 550 °C; gas 1, 60 psi; gas 2, 60 psi (all nitrogen); voltage, 4000 V; collision-activated dissociation (CAD), 12 (arbitrary unit). The Analyst^®^ 1.6 software was applied to evaluate the chromatograms using the internal standard method and peak-area ratios for calculation (quadratic regression, 1/x weighting).

The analytical method was validated with respect to linearity, precision (within-day and between-day), accuracy (within-day and between-day), selectivity, and rack stability regarding the FDA Guidance for Industry “Bioanalytical Method Validation” (Issue: May 2001). All validated criteria met the requirements of the FDA guideline. Lower limit of quantification (LLOQ) was 5 ng/mL determined in the matrix.

#### 2.5.3. Determination of the In Vivo Disintegration Parameters

Similar to the in vitro parameters, two in vivo parameters were chosen to describe the in vivo disintegration. Initial capsule or tablet disintegration (iDT) was defined by reaching a salivary caffeine concentration which exceeded the triple limit of quantification (15 ng/mL). This time point had to be supported by the concentration of the next sampling point, which should be either more or not less than 5% of the concentration value before. This procedure was described in more detail in a recent publication [18]. In order to describe the kinetics of in vivo disintegration, the difference (ΔT_max_) between salivary caffeine T_max_ and iDT was calculated. Generally, a low ΔT_max_ indicated a rapid and complete disintegration, whereas a higher value for ΔT_max_ was an indicator for a slow disintegration of the dosage form.

#### 2.5.4. Statistical Comparison 

The statistical comparison was performed for the two in vivo parameters iDT and ΔT_max_ as one single value for every in vivo experiment between the four groups. As statistical analysis of the experiments reviled non-normality of the observations, nonparametric tests were used. In order to determine if the observations originated from the same distribution, the Kruskal–Wallis test was used. For the direct comparison between the four groups, the Wilcoxon signed-rank test was used. All *p*-values in the manuscript are given based on this test.

## 3. Results

### 3.1. In Vitro Experiments

#### 3.1.1. Compendial Dissolution Testing

**Figure 4:**
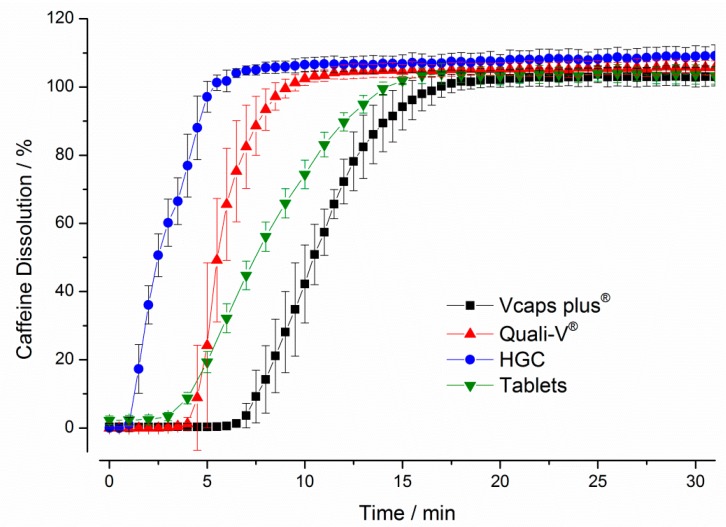
Dissolution of caffeine over time for four immediate release dosage forms in the paddle apparatus. The stirring speed was 75 rpm, the medium temperature was 37 ± 0.5 °C and a total volume of 900 mL simulated gastric fluid pH 1.2 without pepsin was used. The drug concentrations were determined by fiber optic UV measurement at 272 nm (mean ± SD, *n* = 6).

The results obtained with USP paddle apparatus are presented in Figure 4. It can be seen that the hard gelatin capsules (HGC) showed the fastest dissolution with a drug release of more than 80% within 5 min. In contrast, the longest delay in drug release was observed for the Vcaps^®^ plus capsules, for which a drug release of more than 80% was obtained only after 13.5 min. Compared to the HGC, both HPMC capsules showed higher variability during drug release as can be seen from the higher standard deviations.

#### 3.1.2. GastroDuo Experiments

**Figure 5:**
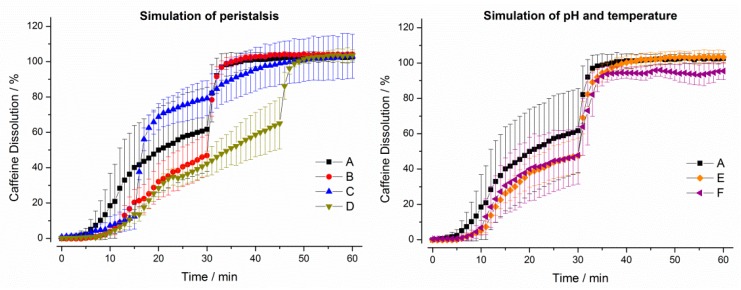
Dissolution profiles of immediate release tablets in the GastroDuo. *Left*: Programs simulating the influence of peristalsis (A–D), *Right*: Programs simulating the influence of temperature and pH value (A,E,F). The concentration was measured in the outflow of the gastric cell by fiber optic UV measurement (mean ± SD, *n* = 6).

As can be seen from Figure 5, the drug release profiles measured by using the GastroDuo were different to the profiles obtained by using the USP paddle apparatus. In the following sections, we will briefly describe the results of the in vitro experiments for every formulation.

For the IR tablet, drug release was not complete in any of the six programs until the simulation of gastric emptying. In test program A, only around 60% of the drug was released until this time point. Notably, the initial stress event had only minor effects on drug release. In programs B and D, drug dissolution was again slower due to lower mechanical (pressure) and hydrodynamic (flow rates) stresses in the first 30 min. The simulation of constant pH and temperature did not lead to relevant differences in drug dissolution.

**Figure 6:**
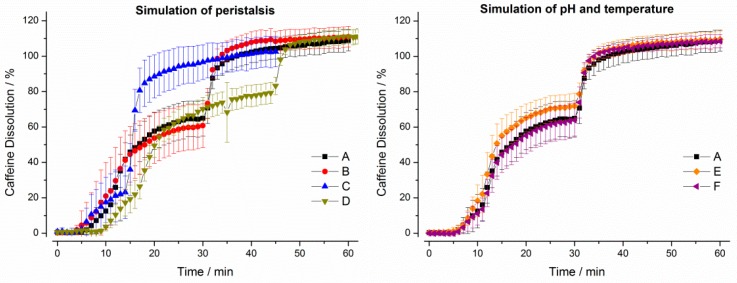
Dissolution profiles of Quali-V^®^ capsules in the GastroDuo. *Left*: Programs simulating the influence of peristalsis (A–D), *Right*: Programs simulating the influence of temperature and pH value (A,E,F). The concentration was measured in the outflow of the gastric cell by fiber optic UV measurement (mean ± SD, *n* = 6).

For Quali-V^®^ capsules, only small effects of simulated peristalsis as well as of pH and temperature on drug release were observable in the in vitro experiments (Figure 6). The profile was mainly dictated by the process of gastric emptying.

**Figure 7:**
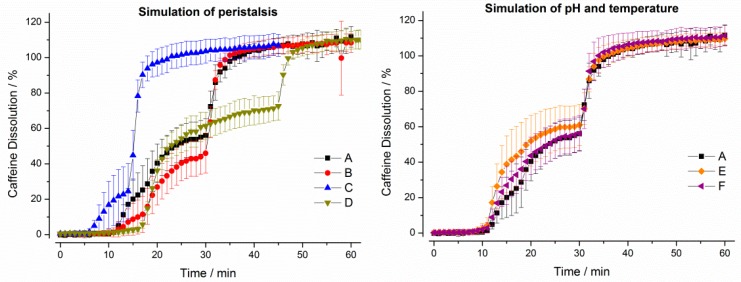
Dissolution profiles of Vcaps^®^ plus in the GastroDuo. *Left*: Programs simulating the influence of peristalsis (A–D), *Right*: Programs simulating the influence of temperature and pH value (A,E,F). The concentration was measured in the outflow of the gastric cell by fiber optic UV measurement (mean ± SD, *n* = 6).

It can be seen from Figure 7 that the simulation of mechanical and hydrodynamic stresses was important for drug release from Vcaps^®^ plus. Whereas the short gastric transit time in program C led to a fast dissolution of the caffeine, drug release was slow in test programs B and D, where lower mechanical stresses were present. The simulation of pH and temperature profiles did not lead to any relevant changes.

**Figure 8:**
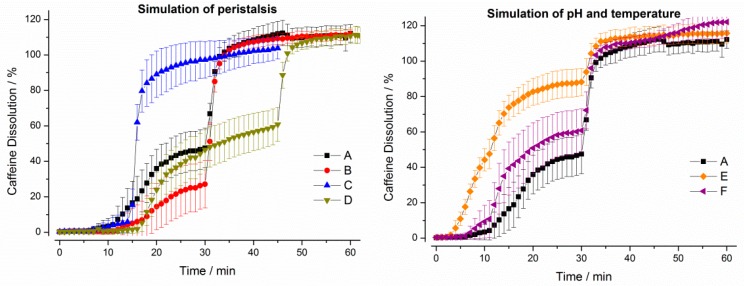
Dissolution profiles of hard gelatin capsules in the GastroDuo. *Left*: Programs simulating the influence of peristalsis (A–D), *Right*: Programs simulating the influence of temperature and pH value (A,E,F). The concentration was measured in the outflow of the gastric cell by fiber optic UV measurement (mean ± SD, *n* = 6).

It can be seen from Figure 8, that the constant higher temperature of 37 °C (program E) as well as the constantly low pH of pH 1.2 (program F) in the gastric cell accelerated drug release from the hard gelatin capsules. Moreover, dissolution was also accelerated in programs characterized by higher mechanical and hydrodynamic stresses in the first 30 min (A and C).

**Figure 9:**
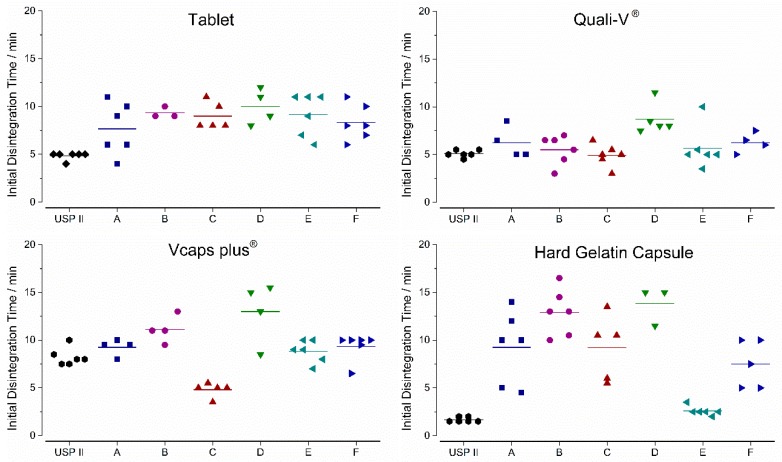
In vitro initial disintegration times (iDT) of four immediate release dosage forms. Given for all six test programs of the GastroDuo and the compendial dissolution. Diamonds indicate individual values and solid lines indicate means.

The initial disintegration times (iDT) are given in Figure 9. For the USPII data iDT was based on the individual dissolution profiles, whereas GastroDuo data was based on the caffeine concentrations measured in the outflow of the gastric cell. The highest variability in between the test programs was observed for the HGC ranging from 2.5 to 17 min, whereas Quali-V^®^ and the film-coated tablet exhibited a lower variability ranging from 3 to 12 min. The lowest variability between test program A was observed for Vcaps^®^ plus.

### 3.2. In Vivo Experiments

**Figure 10:**
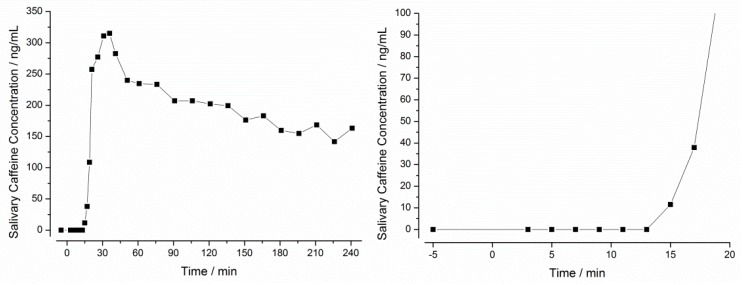
Salivary caffeine concentration over time after administration of a Vcaps^®^ plus capsule with 25 mg caffeine to one healthy volunteer after a 10 h fasting period. *Left*: Whole observation time frame. *Right*: Detail of the first 20 min.

In all 14 subjects, caffeine concentration-time profiles were successfully measured in the saliva. These profiles were used to calculate the initial disintegration time (iDT) and the ΔT_max_. An exemplary caffeine saliva concentration-time profile is given in Figure 10. 

In this example, the first caffeine concentration above LLOQ (5 ng/mL) was observed after 15 min (11 ng/mL). As described earlier, the definition of the iDT was based on a concentration value at least three times above LLOQ (15 ng/mL). Since the first concentration above this value was measured after 17 min, this time was defined as iDT. Since Salivary caffeine t_max_ in this example was observed after 36 min, a ΔT_max_ of 19 min was calculated.

**Figure 11:**
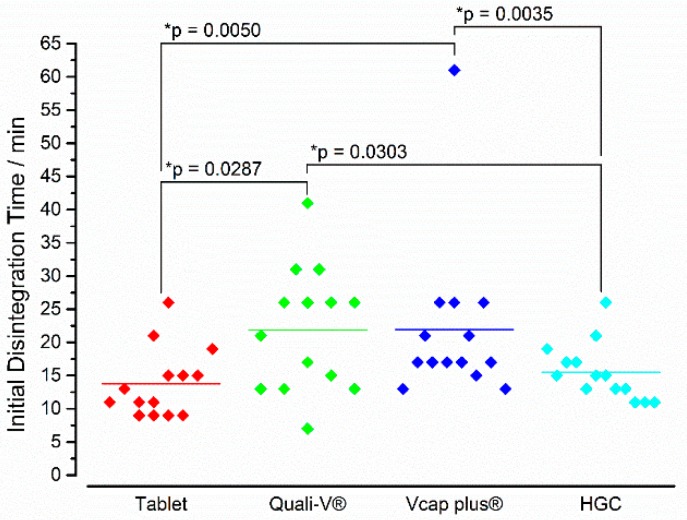
In vivo iDT of the four immediate release dosage forms. Diamonds indicate individual values and solid lines indicate means. The * *p*-value was based on the two-tailed Wilcoxon signed-rank test (*n* = 14), *p* < 0.05 indicating a significant difference.

In Figure 11, the iDTs of all four formulations are depicted. Interestingly, the highest variability was observed for Quali-V^®^ and Vcaps^®^ plus capsules. These two IR formulations also showed the highest mean initial disintegration times (>20 min). In between these two formulations, a statistically significant difference could not be observed. In contrast, the tablets (13.8 ± 5.2 min) and the hard gelatin capsules (15.5 ± 4.3 min) started to disintegrate earlier with no statistical difference between these two dosage forms. Notably, between these two groups (i.e., capsules based on HPMC and tablets/hard gelatin capsules) statistically significant differences were present.

**Figure 12:**
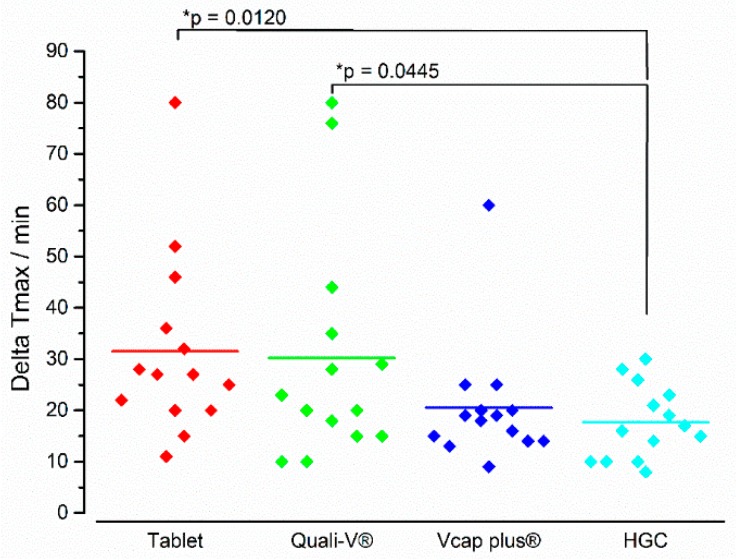
In vivo ΔT_max_ of the four immediate release dosage forms. Diamonds indicate individual values and solid lines indicate means. The * *p*-value was based on the two-tailed Wilcoxon signed-rank test (*n* = 14), *p* < 0.05 indicating a significant difference.

With respect to the kinetics of the in vivo disintegration process, a different picture was seen for ΔT_max_ than for iDT (Figure 12). Whereas the tablet showed the fastest iDT, it had the longest ΔT_max_. The lowest ΔT_max_ was seen for HGC and this value was significantly lower than for the tablet and the Quali-V^®^ capsule. It has to be noted, that with the except of the HGC, all formulations showed individual values diverging around 40–50 min to the mean value.

## 4. Discussion

In this work, the disintegration and dissolution behavior of four different IR formulations were investigated in vitro by using a novel dissolution tool, the GastroDuo, and in vivo by using the salivary tracer technique. The four formulations tested in this work included one tablet formulation and three different capsule formulations. They were all based on the same powder mixture composed of caffeine, lactose monohydrate, and croscarmellose in order to directly compare the behavior of the formulations and to minimize the effect of further parameters (e.g., certain excipients, particle size, etc.). The powder mixture was filled manually into three different capsule shells that were based either on gelatin (hard gelatin capsules) or HPMC (Quali-V^®^, Vcaps^®^ plus). In case of the tablets, the powder mixture was compressed to tablets on a rotary tablet press and coated with HPMC (5% weight gain) in a subsequent step.

Compendial dissolution testing with the paddle apparatus revealed that the hard gelatin capsule disintegrated first, whereas Quali-V^®^ capsules had the longest lag time until beginning of disintegration. In case of the tablets and the Vcaps^®^ plus capsules, the starting point of disintegration was comparable for both formulations, but subsequently the capsules released their content faster. This may be due to the fact that after the capsule shell ruptured, the particles were released and, thus, presented a larger surface area available for drug dissolution. In line with this hypothesis, we also observed similar dissolution rates for the other two capsule shells.

In contrast to compendial dissolution test methods, the GastroDuo was able to simulate variable gastric emptying kinetics, dynamic pH, and temperature profiles as well as gastric peristalsis. The basis for these simulations were recent in vivo studies that were performed with healthy subjects [3,4,6,19]. For instance, the application of MRI allowed us to describe the kinetics of gastric emptying in high temporal resolution [6], whereas telemetric capsules such as SmartPill provided deeper insights into luminal conditions in terms of pH value, temperature, and pressures [3]. In order to reflect the large variability of these physiological parameters in the fasted state, six test programs were defined. Thereby, program A represented the expected ‘average’ profile: gastric emptying of water following first-order kinetics within 30 min, an early pressure event of 200 mbar after 10 min, and the presence of MMC phase III activity after 30 min. The other five programs represented modifications of single parameters in order to cover the extremes that may occur in the human stomach. By this, the variability of drug release that may occur in vivo should be investigated. 

The GastroDuo experiments demonstrated that the simulated physiological variability of the different parameters was highly important for the disintegration behavior of the four formulations. Compared to what has been observed with compendial dissolution testing, the starting point of disintegration was clearly later for all formulations in the GastroDuo. This effect could be explained by the mild conditions inside the GastroDuo in terms of shear rates and stresses that were simulated in the GastroDuo. In contrast, the high shear rates applied in the paddle apparatus overestimated the disintegration process of the different formulations as was shown later in vivo. In test program A, which was regarded as the standard program, the disintegration times were more or less comparable with values between 5 and 10 min. However, especially in case of the hard gelatin capsule the disintegration times were highly variable with values ranging from 5 to 15 min. At the end of simulated gastric transit, around 40%–60% of the drug had been transferred to the acceptor vessel.

As can be seen from the comparison of the results of test programs A and B, the presence of an initial pressure event was highly relevant especially for the hard gelatin capsules and for the tablets but was of minor importance for the HPMC-based capsules. Apparently, only small pressures were needed to initiate the disintegration of the formulations. The presence of single pressure events has been observed also in recent in vivo studies and their timing may contribute significantly to the in vivo variability. In test programs C and D, the pressure event was simulated after 5 and 15 min, respectively. Interestingly, it was only in case of Vcaps^®^ plus that an earlier pressure event also caused an earlier starting point of disintegration. For the other formulations, this event was of minor importance. This effect may have to do with penetration of water into the tablet and with the swelling of the polymer in case of the capsule shells. Apparently, 5 min was not sufficient to cause a relevant softening of the dosage form. Thus, they could withstand the simulated pressure. The simulation of a later pressure event in program D initiated the disintegration of Vcaps^®^ plus capsules and of hard gelatin capsules. In case of the tablets and the Quali-V^®^ capsules, disintegration started before this event and, thus, the simulation of this pressure event only accelerated drug release by improving mixing of released contents. Thus, peristaltic contractions are not only important to initiate disintegration of the dosage form, but also to allow mixing of undissolved particles which will facilitate dissolution. Analogous effects have been observed for hydrogel matrix tablets in combined pharmacokinetic and magnetic marker monitoring studies. The drug release rate of felodipine matrix tablets was shown to be highly dependent on the intragastric localization. An onset of drug plasma concentrations could not be observed as long as tablets were located in the fundus [20]. This effect can also be explained by the absence of peristaltic contractions. In all programs and for all formulations, the simulation of intense gastric peristalsis and of with high flow rates, by which we simulated the emptying of any residues from the stomach during MMC phase III, resulted in the rapid increase of caffeine levels in the acceptor vessel. This highlights the importance of the MMC for the onset of drug plasma concentrations after oral drug administration in fasted state. The same effect has been recently demonstrated in vivo by van den Abeele and colleagues [21].

As the timing of MMC phase III activity with respect to the time point of drug administration appears to be highly relevant, we simulated different gastric transit times [21]. Especially for short gastric transit times, the contribution of this final event to the overall drug transfer to the acceptor vessel was great for all formulations. For the hard gelatin capsule, more than 90% of the caffeine was released as a result of an early phase III activity. In contrast, prolonged gastric transit times (program D) resulted in different effects. In the case of the tablets, decreased transfer rates also resulted in a delayed onset of caffeine levels in the acceptor vessel. On the contrary, the onset of caffeine levels in the acceptor vessel was similar to the standard program for the capsule formulations. Thus, at the end of the longer simulated gastric transit (45 min), a higher amount of caffeine was present in the acceptor vessel compared to the standard program.

Recent studies have already shown that the simulation of temperature profiles is of high relevance for hard gelatin capsules since the gelatin starts to dissolve only above temperatures of 32 °C [15]. Therefore, the simulation of physiologically pH and temperature profiles was regarded as crucial for the development of the biorelevant dissolution tool presented herein. In line with the aforementioned study, we could show that the temperature dip which is caused by the intake of an oral formulation together with a glass of water at room temperature delays the disintegration of hard gelatin capsules. As can be seen from the results of program E but also from the compendial dissolution experiment, disintegration occurs earlier if the temperature is constantly at 37 °C. For the other formulations, the temperature of the medium was only of minor importance. Similar effects were observed for the simulated pH profile as neither the caffeine nor the used excipients experienced a pH-dependent dissolution behavior. Drug release from the tablets was slightly slower but this effect was not considered relevant.

The dissolution experiments performed with the GastroDuo nicely revealed that drug release even from very relatively simple formulations of a highly soluble drug can be influenced to different extents by various physiological parameters. The four IR formulations investigated in this study clearly showed that the simulation of peristalsis strongly affected in vitro drug release. These effects showed varying tendencies in between the formulations, although they were generally considered as comparable. Furthermore, the GastroDuo was able to discriminate between formulations, which was not possible in compendial experiments. The next step was the investigation whether these differences could be confirmed in vivo.

The salivary tracer technique represented an interesting tool to check the in vivo relevance of the data that were obtained by using the GastroDuo. In a recent study, it has been confirmed by MRI that this technique can be used to determine in vivo disintegration times with an acceptable accuracy [18]. For this reason, we determined the disintegration behavior of the four formulations in 14 healthy subjects by using salivary caffeine concentrations. In comparison to the results from scintigraphic studies [22,23,24,25,26], in which the starting point of disintegration of hard gelatin capsules was typically in the range of 4–12 min, the mean iDT for HGC in this study was 16 ± 4 min and, thus, clearly longer. Similar effects were also observed for the other capsules. This difference can be at least partly explained by the fact that scintigraphy is a direct imaging method, whereas the salivary tracer technique requires additional processes such as dissolution, gastric mixing, and emptying of the tracer. These processes probably caused an offset between the disintegration of the formulation and the appearance of caffeine in saliva. In a recent study, the offset between the salivary tracer method and MRI as a direct imaging method was on average approximately 5 min [21]. Due to this limitation, the initial disintegration times determined in vitro and in vivo cannot be compared directly. However, the in vivo data could be used to rank-order the four formulations as the offset was expected to be independent from the formulation.

In terms of the starting point of in vivo disintegration, two groups could be identified between which a significant difference could be detected. The starting point of disintegration of the tablets and the hard gelatin capsules was after around 15 min, whereas the HPMC-based capsules began to disintegrate on average only after more than 20 min. Within these groups, i.e., between tablets and HGC as well as between Quali-V^®^ and Vcaps^®^ plus, we could not find statistically significant differences. Especially in case of Quali-V^®^ capsules, these data were in contrast to the in vitro data obtained with the GastroDuo. Based on the GastroDuo data, we would have expected that the disintegration of Quali-V^®^ capsules occurs earlier or at similar time points compared to the tablet or the HGC. One reason for this effect might have been the gastric peristalsis again. For the sake of reproducibility, the blades within the gastric cell performed slow movements every 3 min. However, the mechanical forces that were caused by these movements may have been high enough to initiate the rupture of the capsule shell of the HPMC based formulations. This sensitivity towards small mechanical pressures may also explain the large variability that was seen in vivo. Another explanation may be based on the special characteristics of the gelling agent, which was carrageenan in case of the Quali-V^®^ capsules. The sulphate group of carrageenan is known to interact with polar substances, even in acidic media. This interaction can cause a delayed disintegration of the capsule shell, which may have occurred in vivo [27,28]. In contrast to the observations made in this study, Tuleu and colleagues determined disintegration times of 9 ± 2 min for Quali-V^®^ capsules with the aid of scintigraphy [24]. Thus, further studies will be needed to study this effect in more detail.

In general, the in vitro data obtained with the GastroDuo provided a good impression of the in vivo situation, but with some limitations. One of the most surprising in vivo observations was the relatively low variability of the in vivo iDT of the hard gelatin capsules. Based on the in vitro dissolution experiments, a higher variability compared to the other IR formulations was expected. An explanation for this overestimation of variability in the GastroDuo was the weighting of the six test programs. It should be noted that four of the six test programs were mimicking physiological extrema (low and high peristalsis, constant pH, constant temperature, etc.) which are not always likely to occur in a such relatively small number of subjects. Based on the in vivo observations, we would emphasize that the test programs A and D are more likely to occur than the other test programs. On the other hand, under the controlled conditions of BE/BA studies, a situation like in program E (constant gastric temperature of 37 °C) is rather unlikely. However, in terms of real-life variability, such a case may be likely, for instance, when a patient administers a drug product with a warm or hot drink such as coffee or tea. Similar effects could be observed with respect to motility and gastric emptying kinetics. Grimm and colleagues showed recently that gastric emptying of 240 mL of water can take between 10 and 50 min [6]. Thus, it is likely that several individuals in this study showed gastric emptying times that were outside the range simulated in vitro (i.e., 15–45 min). With these considerations in mind, the dissolution results should be interpreted not only based on the data but also with respect to the likelihood of each test program to occur in vivo. 

Interestingly, we also observed in vivo disintegration times of 41 or 61 min in this study. Such long in vivo disintegration can be caused by various factors. Apart from a lack of mechanical stresses acting on the dosage forms, such long times can also be caused by exceptionally long esophageal transit times. The retention of capsules in the esophagus was reported in the past for hard gelatin and also for HPMC capsules [29,30,31]. Furthermore, the localization of the dosage form in the stomach can be variable. As the gastric emptying of fluids can be relatively fast, it is possible that dosage form localization in areas with limited fluid volumes may hamper its disintegration. Additionally, the floating of the formulation on top of the gastric contents, which is typically observed in vivo, as well as sticking to the stomach wall can also contribute to the observed in vivo variability of disintegration. 

For the delivery of drugs by immediate release formulations the time point of the beginning of disintegration is not the only relevant parameter; it is also the subsequent phase of drug release, especially for drugs which are subject to a concentration dependent absorption [32]. A rapid and complete drug release typically results in higher concentrations, which can drive absorption and, thus, lead to changed pharmacokinetics compared to a slow and stepwise disintegration. A straightforward parameter to describe the kinetics of drug release from an IR formulation in this study was the time difference between t_max_ and the beginning of disintegration (ΔT_max_). A small value indicated a rapid drug release whereas larger values indicated slower drug release. With mean values of 31 and 30 min for ΔT_max_, the tablet and Quali-V^®^ exhibit the slowest drug release. In contrast HGC showed a very fast and reproducible dissolution within 18 min. Vcaps^®^ plus released the drug slightly slower within 20 min but with a higher variability. The in vivo data revealed that although Vcaps^®^ plus and Quali-V^®^ started to disintegrate at similar times, Vcaps^®^ plus showed a mean ΔT_max_ that was 6 min shorter compared to Quali-V^®^. One likely explanation for this difference is that the disintegration of the capsule shell must have been different. The highest ΔT_max_ was reported for the tablet, which could be explained by the fact that tablet disintegration is typically based on a more or less continuous erosion, whereas in case of the capsule, the powder was released within a relatively short time and subsequently, presented a large surface available for drug dissolution. However, it must be considered that ΔT_max_ was based on salivary caffeine t_max_, and, thus, certainly influenced by gastric emptying. It cannot be excluded that the formulation disintegrated fully in the stomach, but the released content was not emptied completely into the small intestine. It is generally possible that larger parts of the drug are retained in the stomach, especially if the coadministered water was already emptied. In this case, t_max_ would depend largely on the occurrence of the phase III activity of the MMC. Hence, we would underestimate the rate of drug release. Nonetheless, this effect was assumed to be similar for all four formulations that were investigated in this controlled cross-over study.

In contrast to human GI physiology, the GastroDuo is a more rigid system that allows a certain level of standardization which is needed for dissolution experiments. This standardization of the test programs has the advantage that the conditions can be fully controlled and changed independently from each other. This allows the identification of crucial parameters for both disintegration and dissolution of an oral formulation early on. Thus, the GastroDuo should always be regarded as a compromise between a physiologically relevant simulation of the in vivo situation and a fully controlled dissolution test system which allows the monitoring of all relevant parameters.

## 5. Conclusions

In this work, the GastroDuo was applied to study the disintegration and drug release behavior of four different IR formulations in the fasted stomach under physiologically relevant conditions. This novel dissolution tool enabled a realistic simulation of gastric emptying, gastric motility, as well as luminal pH and temperature profiles. With the aid of six test programs, the physiological range of these parameters should be covered. The results of this study revealed that the GastroDuo was able to detect certain differences between the tested IR formulations, which could also be confirmed in an in vivo study conducted in healthy volunteers by using the salivary tracer technique. However, compared to in vivo data, the in vitro variability was larger for certain formulations. This suggests that not all test programs were likely to occur under the controlled and standardized situation of clinical trials. It should be considered that some of them have represented physiological extremes which may occur only in certain patient populations or under certain conditions. However, they allow to study the sensitivity of certain formulations towards individual physiological factors. Therefore, the GastroDuo represents a valuable tool to understand disintegration and dissolution of oral dosage forms.

## Figures and Tables

**Table 1:** Dosage forms used in the present study.

Dosage Form	Ingredients
**Immediate release tablets with 5% HPMC coating**	Caffeine, croscarmellose, lactose, magnesium stearate, silica dioxide, hydroxypropyl methylcellulose
**Hard gelatin capsule (size 0)**	Caffeine, croscarmellose, lactose, gelatin
**Quali-V^®^ capsules (size 0)**	Caffeine, croscarmellose, lactose, hydroxypropyl methylcellulose, carrageenan
**Vcaps^®^ plus capsules (size 0)**	Caffeine, croscarmellose, lactose, hydroxypropyl methylcellulose

**Table 2:** Demographic data of the study volunteers.

Age	22–31 years
Body Mass Index	20–25
Female/Male	6/8
Ethnics	Caucasian
Pre-existing conditions	none
Number of volunteers	14
Regular coffee consumers	12

## Supplementary Materials

The following are available online at https://www.mdpi.com/1999-4923/11/12/659/s1. The video file “GastroDuo_Video.MOV” can be found in the supplementary materials.
